# Non-Woven Sheet Containing Gemcitabine: Controlled Release Complex for Pancreatic Cancer Treatment

**DOI:** 10.3390/polym14010168

**Published:** 2022-01-01

**Authors:** Kazuma Sakura, Masao Sasai, Takayuki Mino, Hiroshi Uyama

**Affiliations:** 1 Respiratory Center, Osaka University Hospital, Osaka 565-0871, Japan; 2 Department of Surgery, Graduate School of Medicine, Osaka University, Osaka 565-0871, Japan; 3 Division of Translational Research, Osaka University Hospital, Osaka 565-0871, Japan; sasai-masao@tissue.med.osaka-u.ac.jp; 4 Department of Applied Chemistry, Graduate School of Engineering, Osaka University, Osaka 565-0871, Japan; t.mino.OU2009@gmail.com (T.M.); uyama@chem.eng.osaka-u.ac.jp (H.U.)

**Keywords:** pancreatic cancer, gemcitabine, controlled release, non-woven sheet, chemotherapy, antitumor efficacy, poly(L-lactic acid)

## Abstract

The 5-year survival rate for pancreatic cancer remains low, and the development of new methods for its treatment is actively underway. After the surgical treatment of pancreatic cancer, recurrence and peritoneal dissemination can be prevented by long-term local exposure to appropriate drug concentrations. We propose a novel treatment method using non-woven sheets to achieve this goal. Poly(L-lactic acid) non-woven sheets containing gemcitabine (GEM) were prepared, and GEM sustained release from this delivery system was investigated. Approximately 35% of the GEM dose was released within 30 d. For in vitro evaluation, we conducted a cell growth inhibition test using transwell assays, and significant inhibition of cell growth was observed. The antitumor effects of subcutaneously implanted GEM-containing non-woven sheets were evaluated in mice bearing subcutaneous Panc02 cells, and it was established that the sheets inhibited tumor growth for approximately 28 d. These results suggest the usefulness of GEM-containing non-woven sheets in pancreatic cancer treatment.

## 1. Introduction

Pancreatic cancer is difficult to manage, and the 5-year survival rate in patients suffering from the disease is less than 5% [1]. Because it is usually far advanced at the time of diagnosis, even after operative resection, relapse often occurs and is detected as multiple liver metastases, local recurrence, or peritoneal dissemination soon after surgery, resulting in a significantly poor disease prognosis. At present, surgical resection is the first and most effective therapeutic option for the treatment of localized pancreatic cancer without distant metastases; however, after pancreatectomy, 40.1% of patients have positive resection margins [2], 32.6% have local recurrence [3], and 13% develop peritoneal dissemination [4]. 

Therefore, it is important to control local recurrence after pancreatectomy to improve patient survival rates. Although intra-operative radiotherapy as an adjuvant treatment strategy has been used in a bid to reduce local recurrence, its effectiveness is still controversial as a recent randomized trial (ESPAC-1) showed that adjuvant chemoradiotherapy has a negative effect on patient survival [5]. Thus, it is thought that there is no effective local postoperative treatment option for pancreatic cancer. Trans-tissue local therapy has some advantages, such as a high local drug concentration at the target tissue and relatively low concentration in systemic organs, resulting in a significant enhancement of drug therapeutic effects and marked reduction in systemic adverse effects, in both frequency and severity. 

Gemcitabine (GEM) is an anticancer drug classified as “pyrimidine antagonist” and is an antimetabolite. GEM enters the DNA of cancer cells, inhibits the synthesis of DNA required for cell division, eliminates the cancer cells, and suppresses the spreading and growth of the cancer. The cytotoxicity of gemcitabine is not limited to the S phase of the cell cycle and is equally effective in saturated and exponential cells. Toxicity appears to be the result of a combination of several effects on DNA synthesis [6]. In other words, GEM is an anticancer agent whose activity depends on the concentration and the time of contact with cells [7,8,9]. GEM has been used in pancreatic cancer, non-small cell lung cancer, biliary tract cancer, urinary epithelial cancer, unresectable breast cancer, and ovarian cancer that have worsened after chemotherapy [6]. Typical adverse events are myelosuppression, which is a decrease in white blood cells and platelets [6]. Clinical effectiveness of GEM in pancreatic cancer is limited by its rapid plasma metabolism and development of chemo-resistance [10]. To address this concern, we developed non-woven sheets containing GEM to be used in local therapy of pancreatic cancer, featuring a continuous release of GEM.

The treatment of local lesions by subcutaneously introducing an anticancer drug reservoir is a therapeutic strategy for local intraperitoneal cancer. It enables repeated and sustained drug release to a target lesion; however, several risks are associated with this method, such as catheter obliteration, infection via the catheter, and the development of mechanical ileus owing to the presence of the catheter in the abdominal cavity [11]. Some controlled release devices have also been developed for local cancer treatment. A study reported a local pancreatic cancer therapeutic strategy using a mixture of fibrin glue and GEM [12]. However, there are challenges to employing controlled release devices in clinical settings owing to the difficulty of maintaining a steady gradual supply of the drug and using these devices over an extended period of time. We focused on pancreatic cancer treatment and the prevention of its local recurrence and dissemination because chemotherapy for the disease is limited by the difficulty of drug administration after surgery. Anticancer drugs are often poured into the peritoneal cavity in hyperthermal conditions in cases in which cytological dissemination is detected by rapid pathological diagnosis or in those with a high risk of rapid recurrence [13]. However, maintaining the concentration of water-soluble anticancer drugs is difficult. Recently, electrospinning has received considerable scientific interest as a convenient and straightforward process for the fabrication of non-woven sheets of ultrafine fibrous biodegradable polymers [14]. Electrospinning is superior to conventional methods in that it can be conveniently used to fabricate various composite nanofibers that cannot be fabricated using conventional methods [15]. This implies that there is the possibility of fabricating novel local therapeutic devices for use in the human body.

The diameters of electrospun fibers often fall within the sub-micron range, whereas those of conventional polymer fibers are usually greater than the micron range [16]. The small diameter and non-woven morphology of these fibers result in a large specific surface area, which is advantageous for filter and biomedical applications. 

## 2. Materials and Methods

### 2.1. Chemical Materials

Poly(L-lactic acid) (PLLA; H-900, Mn = 61.323, Mw = 123.284, Mw/Mn = 2.010) was purchased from Mitsui Chemicals (Tokyo, Japan). GEM was purchased from Eli Lilly Japan K.K. (Hyogo, Japan). The solvent 1,1,1,3,3-hexafluoro-2-propanol (HFIP) was purchased from Wako Chemicals (Osaka, Japan). 

### 2.2. Cell Lines

Panc02, murine pancreatic cancer cells, were provided by Dr. Bunzo Nakata (Osaka City University Medical School, Osaka, Japan), and NIH-3T3 cells were purchased from RIKEN Bioresource Research Center (Ibaraki, Japan). 

### 2.3. Animals

Female C57BL/6 mice (6–8-week-old), purchased from CLEA Japan (Tokyo, Japan), were kept under standard housing conditions and provided ad libitum access to food and water. Animals used in all experiments were acclimatized for at least one week in the breeding room of the animal experimentation unit of Osaka University Graduate School of Medicine. In vivo experiments were performed following a protocol approved by the Ethics Review Committee for Animal Experimentation of Osaka University Graduate School of Medicine (#21-055-0).

### 2.4. Preparation of GEM-Containing Non-Woven Sheets 

HFIP was added to 1 g of PLLA and 0.1 g of GEM to make a total mass of 10 g. This solution was used as a PLLA solution with a GEM content of 10 wt%. Other solutions with different GEM contents used for the preparation of PLLA non-woven sheets were prepared in the same way. The solutions were dried for 3 h using a desiccator, with an applied voltage of 17 kV, an injection distance of 15 cm, an injection rate of 3 mL/h, a collector rotation speed of 100 rpm, and an injection volume of 4 mL, using electric field spinning equipment (IMC-164E, Imoto Machinery Co., Kyoto, Japan). The fabricated non-woven sheets were observed using a scanning electron microscope (S-3000N, Hitachi High-Tech Corp., Tokyo, Japan) and an ion-sputtering device (E-1010, Hitachi High-Tech Corp., Tokyo, Japan). The accelerating voltage was 15 kV, and the magnification was 4000 times.

### 2.5. Measurement of GEM Release In Vitro

Approximately 10 mg of 10 wt% GEM-containing PLLA non-woven sheet was cut into rectangles and immersed in phosphate-buffered saline (PBS) in a sample tube. The sample tube was placed on a bio shaker and shaken at 100 rpm at 37 °C. At a specific time point (0 h, 1 h, 4 h, 24 h, 7 d, 30 d, or 60 d), the non-woven sheet was removed and transferred to a new sample tube. Chloroform (1 mL) was added to the sample tube to dissolve the sheet, and then 1 mL of water was added to it. After proper stirring, the aqueous layer was drained off and high-performance liquid chromatography (HPLC) measurements were performed. The separation was performed on an TSKgel ODS-80Tm column (Tosoh, Osaka, Japan) using 0.1 M NaCl solution. The solution was passed through a 0.45-μm filter before being transferred to the sample tube. A GEM peak was observed at approximately 14 min of elution. The GEM concentration was calculated based on the peak area. To determine the quantity of GEM released, the measured GEM quantity was subtracted from the initial quantity.

### 2.6. Measurement of GEM Release In Vivo 

Non-woven sheets were implanted into the backs of the mice. After a specified period of time (0 h, 1 h, 4 h, 24 h, 7 d, 30 d, or 60 d), the non-woven sheets were removed and dissolved, and the concentration of GEM in the water was measured using the same procedure used for measuring the in vitro GEM concentration.

Approximately 10 mg of 10 wt% GEM-containing PLLA non-woven sheet was cut out and implanted into the backs of the mice. After wound suturing, the sutures were cut again at 1 h, 4 h, 1 d, 7 d, 30 d, and 60 d, and the non-woven sheets were removed. These sheets were dried at room temperature (approximately 15–25 °C) to remove excess water. Then, 1 mL of chloroform was added to the sheets and stirred vigorously to dissolve them. After incubation at 37 °C for 1 d, 1 mL of water was added to the aqueous layer. GEM was extracted into the aqueous layer by stirring.

### 2.7. In Vitro Cytotoxicity Assay

Non-woven sheets with different GEM contents were cut into circular forms with a diameter of 14 mm and immersed in 70% ethanol before the cell culture experiments were conducted. The seeding densities of Panc02 cells and NIH-3T3 cells were standardized at 1 × 10^4^ cells/well. Then, the plates were removed after every two days and washed with PBS. Floating cells were washed away, and the number of cells that adhered to the non-woven sheet was determined using a fluorescent plate reader (Synergy HT, Biotek Instruments, Inc., Winooski, VT, USA). For the experiment to evaluate growth inhibition through GEM slow release, cells were first seeded in a transwell plate (Transwell Permeable Supports, 0.4-µm with Size 24 Cluster Plate, PC Membrane, Costar^®^, Thermo Fisher Scientific, Waltham, MA, USA) and, after 6 h, the cut-out non-woven sheets were placed on a net.

The number of cells that adhered to the bottom of the plate after 1, 3, 5, and 7 d was determined using the DNA assay method. First, 1 mL of sodium lauryl sulfate (SDS) solution (SDS 20 mg, sodium chloride 0.90 g, trisodium citrate dehydrate 0.44 g, deionized water 100 mL) was added to each well, and the plates were incubated at room temperature (approximately 15–25 °C) for 1 h. Next, 100 μL from each well was dispensed into 96-well plates, and 100 μL of Hoechst solution (Hoechst 33258 20 μL, sodium chloride 0.18 g, trisodium citrate dehydrate 0.09 g, deionized water 20 mL) was added to each well, in order to measure the fluorescence intensity using a plate reader (setting parameters: excitation wavelength (nm) = 360, emission wavelength (nm) = 460, sensitivity = 80).

### 2.8. Antitumor Efficacy of the GEM-Containing Non-woven Sheets in a Subcutaneous Tumor-Bearing Mouse Model

Approximately 10 mg of 10 wt% GEM-containing non-woven sheets was cut and implanted into mouse back subcutaneous tissues. Then, 1 h, 4 h, 1 d, 2 d, and 7 d following wound suturing, tissues around the non-woven sheets (including subcutaneous fat) were excised. To verify that GEM sustained release leads to its transfer to surrounding tissues without being metabolized, we measured its concentrations in the tissues. As a control for the non-woven sheet, a GEM solution was injected into mouse subcutaneous tissue at a dose of 1 mg (20 mg/mL × 50 μL) per animal. GEM concentrations in the tissues of these control mice were measured at the same time points as those for the non-woven sheet-injected mice.

The weights of the excised tissues were measured, and saline was added to them at a weight several times greater so that the total volume was at least 0.6 mL. Then, the tissue suspension was homogenized using an ultrasonic homogenizer and stored in a refrigerator at −20 °C. GEM tissue concentration measurement was performed at SRL Inc. (Tokyo, Japan) using HPLC.

One million panc02 cells were injected into the dorsal region of C57BL/6 mice. One week after the cell injection, the tumor size reached approximately the size of a grain of rice (a length of approximately 5 mm). After confirmation of the tumor, 10 mg of 10 wt% GEM-containing PLLA non-woven sheet was implanted into mouse subcutaneous tissues. Similarly, as a control experiment, a GEM solution was injected into mouse subcutaneous tissues using a syringe (20 mg/mL × 50 μL). Then, tumor size was measured from the body surface in the days following wound suturing. For tumor size measurement, the long and orthogonal diameters, “a” and “b,” respectively, of the tumors were measured using a ruler. Tumor size was determined using the formula:

Tumor size (mm^3^) = ab^2^/2 (1)

Tumor size was measured from the body surface after every 7 d, starting from the day of implantation of the non-woven sheet.

For the control group (n = 6), approximately 10 mg of GEM-free PLLA non-woven sheet was implanted into mouse subcutaneous tissue, whereas for the systemic group (n = 4), 50 μL of GEM aqueous solution was intraperitoneally administered to the mice at a concentration of 20 mg/mL; for the “GEM aqueous solution group” (n = 4), approximately 10 mg of GEM-free PLLA non-woven sheets and 50 μL of GEM aqueous solution at a concentration of 20 mg/mL were subcutaneously injected into the mice (GEM 1 mg/head). For the “GEM 0.5 mg-containing non-woven sheet group” (n = 4), approximately 5 mg of 10 wt% GEM-containing PLLA non-woven sheets was implanted into the mice (GEM 0.5 mg/head); for the “GEM 1.0 mg-containing non-woven sheet group” (n = 6), approximately 10 mg of 10 wt% GEM-containing PLLA non-woven sheets was implanted into the mice (GEM 1.0 mg/head).

### 2.9. Statistical Analyses

Student’s t-test was used to determine statistical significance, and p-values less than 0.05 were considered statistically significant. All results are expressed as the mean ± standard error of the mean (SEM). 

## 3. Results

### 3.1. Preparation of GEM-Containing Non-woven Sheets

**Figure 1:**
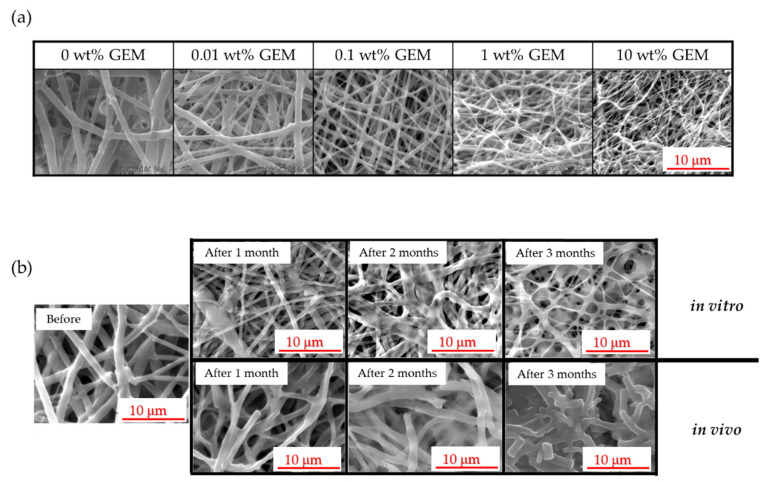
Scanning electron micrographs of GEM-containing non-woven sheets. (**a**) Scanning electron microscopy images of GEM-free and GEM-containing non-woven sheets at concentrations ranging from 0.01% to 10% content. Scale bar: 10 μm. (**b**) Scanning electron microscopy images of denatured GEM-free non-woven sheets after 1, 2, and 3 months of in vitro or in vivo evaluation. Scale bar: 10 μm.

Figure 1 shows the different non-woven sheets prepared at different GEM concentrations, as observed by scanning electron microscopy (Figure 1a). The degradability of the GEM-containing non-woven sheets was evaluated for three months under in vitro and in vivo conditions (Figure 1b). 

### 3.2. Measurement of GEM Release from GEM-Containing Non-woven Sheets

**Figure 2:**
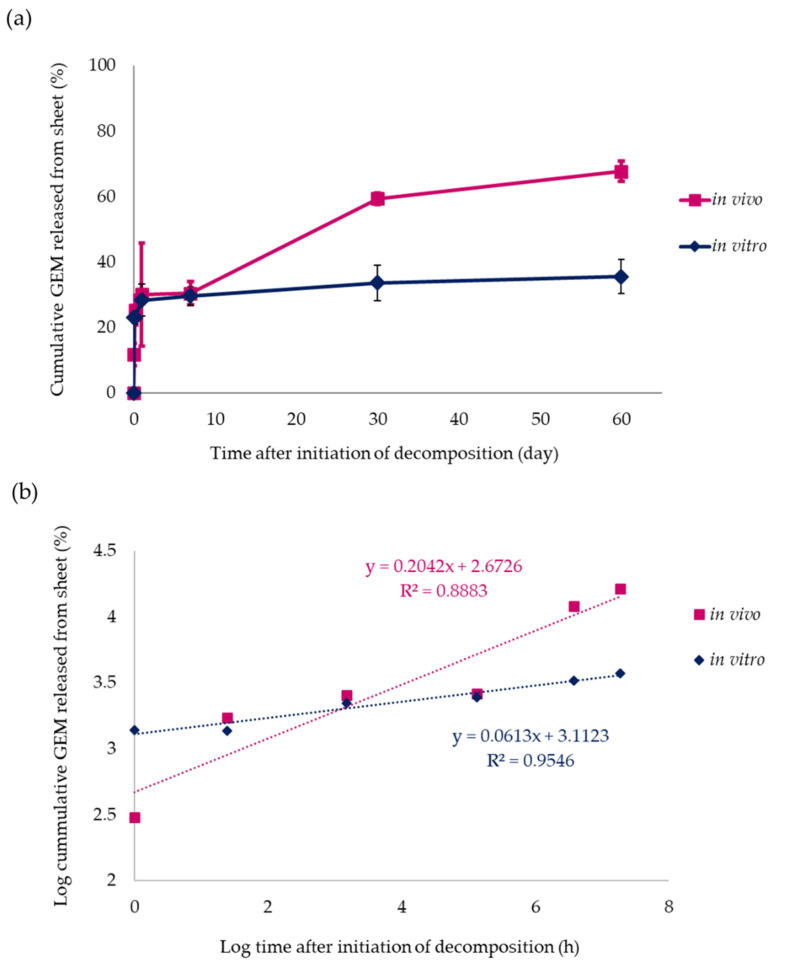
Percentage of GEM released from non-woven sheets with time in vitro and in vivo. (**a**) Cumulative release of GEM from 10 mg of 10 wt% GEM-containing non-woven sheets in vitro and in vivo was evaluated for a period of up to 60 d after non-woven sheet fabrication. After 60 d, 35.6 ± 2.1% and 67.8 ± 1.5% of GEM was cumulatively released in vitro and in vivo, respectively. (**b**) Korsmeyer–Peppas model: the approximate in vitro and in vivo expression levels are calculated as y = 0.0613x + 3.1123 (R^2^ = 0.95) and y = 0.20427x + 2.6726 (R^2^ = 0.89), respectively. Data are presented as the mean ± standard error of the mean (SEM).

Analysis of in vitro GEM release from the non-woven sheets showed an initial burst of 28.4 ± 2.0% on day 1, followed by a 29.8 ± 1.1% and 35.6 ± 2.1 % release by days 7 and 60, respectively (Figure 2a). For In vivo evaluation, the non-woven sheets implanted in the backs of mice were removed after some time, and the quantity of GEM released was determined by measuring GEM concentrations in the non-woven sheets. As in the in vitro evaluation, there was an initial burst of 30.1 ± 7.9 % on day 1, followed by a 30.5 ± 1.5% and 67.8 ± 1.5% release by days 7 and 60, respectively (Figure 2a).

### 3.3. In Vitro Cytotoxicity Assay 

**Figure 3:**
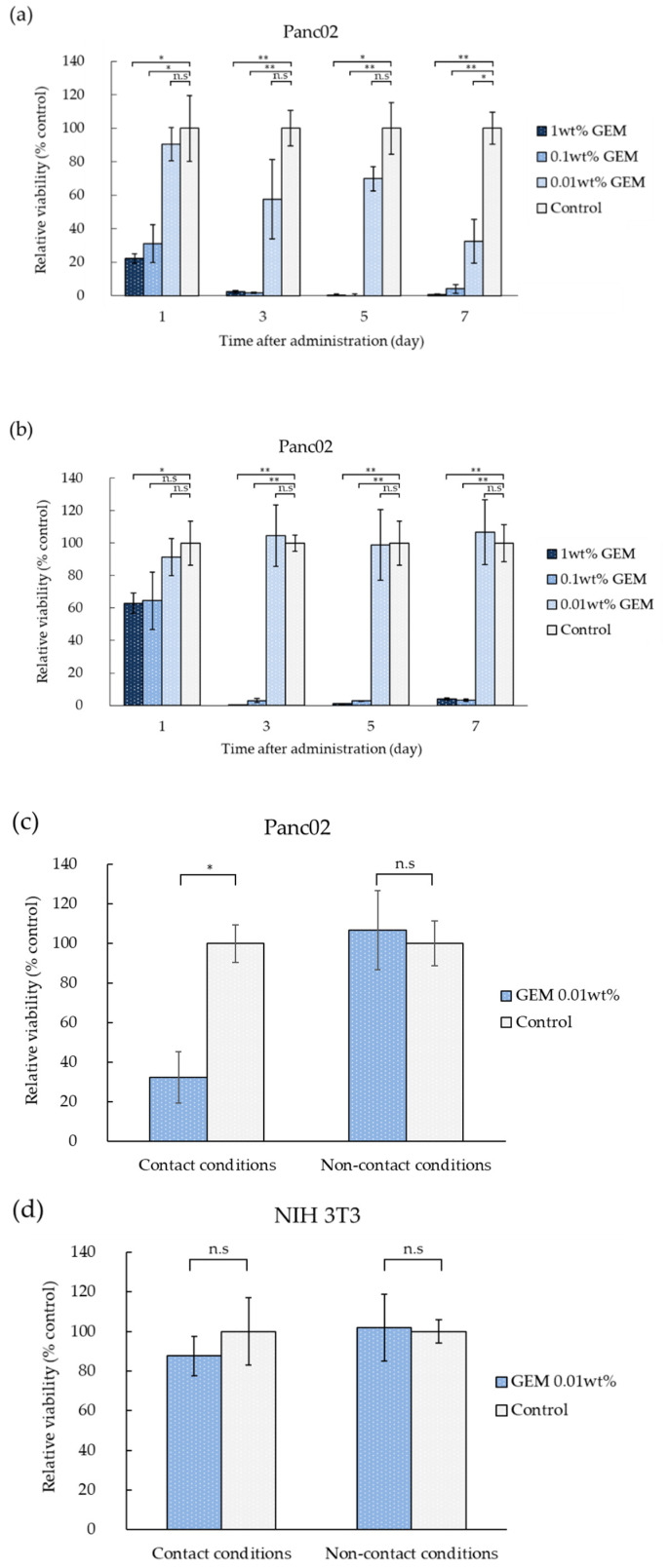
Cytotoxic effects of the non-woven sheets on the murine pancreatic cancer cell line (Panc02) and NIH-3T3 cells. (**a**) Relative cell viability in the treatment group over time (compared with the control group [GEM-free non-woven sheet]). Cell viability on days 1, 3, 5, and 7, respectively, was 22.3 ± 2.8%, 2.3 ± 0.9%, 0.05 ± 0.9%, and 0.6 ± 0.6% in the presence of 1 wt% GEM; 31.1 ± 9.9%, 1.8 ± 0.3%, −5.0 ± 5.8%, and 4.1 ± 2.6% in the presence of 0.1 wt% GEM; and 90.4 ± 9.9%, 57.7 ± 23.7%, 69.9 ± 7.2%, and 32.5 ± 13.0% in the presence of 0.01 wt% GEM (in decreasing GEM concentrations). (**b**) Relative cell viability in the treatment group over time (compared with the control group). Cell viability on days 1, 3, 5, and 7 was 62.9 ± 6.2%, 0.4 ± 0.1%, 1.0 ± 0.1%, and 4.0 ± 0.6% in the presence of 1 wt% GEM; 64.5 ± 17.8%, 3.0 ± 1.2%, 2.7 ± 0.1%, and 3.2 ± 0.6% in the presence of 0.1 wt% GEM; and 91.3 ± 11.4%, 104.6 ± 19.0%, 98.9 ± 21.9%, and 106.8 ± 19.9% in the presence of 0.01 wt% GEM, respectively (in decreasing GEM concentrations). (**c**) Cytotoxic effects of non-woven sheets containing 0.01 wt% GEM against Panc02 cells under contact and non-contact conditions. Non-woven sheets containing 0.01 wt% GEM exhibited significantly higher cytotoxic effects than the control under contact conditions. (**d**) Cytotoxic effects of non-woven sheets containing 0.01 wt% GEM against NIH-3T3 cells under contact and non-contact conditions. Data are presented as the mean ± standard error of the mean (SEM). Significant differences were determined using the Student’s *t*-test (* *p* < 0.05; ** *p* < 0.01; n.s: Not significant).

The evaluation of the cytotoxic activity of the non-woven sheets using a DNA assay showed that cells that were directly attached to the sheets exhibited high GEM concentration-dependent cytotoxicity (Figure 3a). The results of the DNA assay under non-contact conditions, using the transwell plate, showed that only non-woven sheets containing high GEM concentrations exhibited cytotoxic activity; sheets containing 0.01 wt% GEM showed no cytotoxic activity, even on day 7 (Figure 3b).

### 3.4. Antitumor Efficacy of GEM-Containing Non-woven Sheets in Panc02 Tumor Cell-Bearing Mice

**Figure 4:**
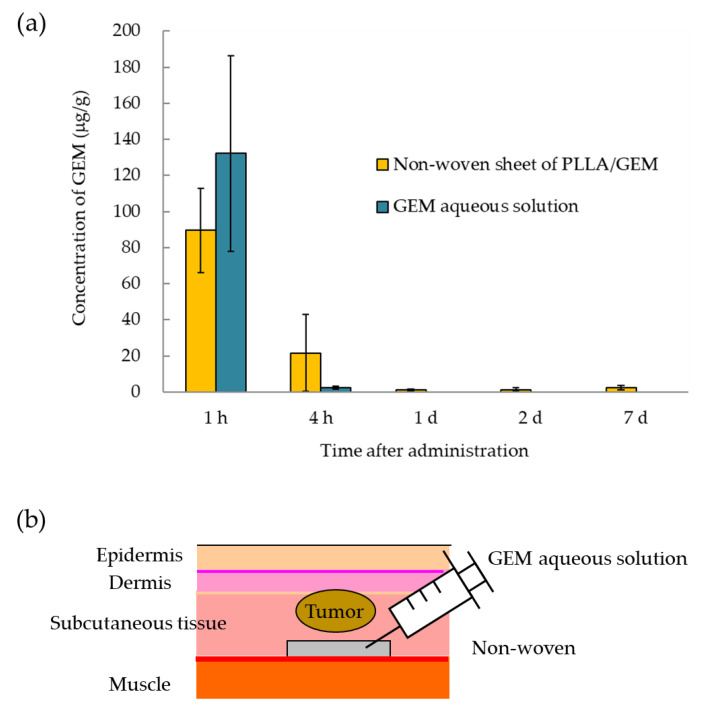

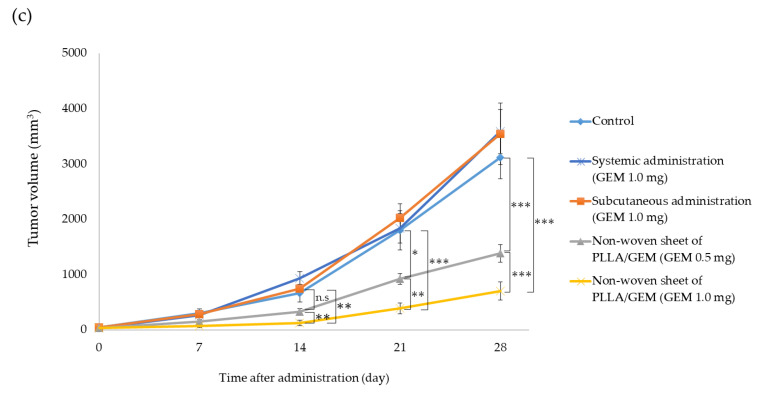
In vivo antitumor effects of the GEM-containing non-woven sheets. (**a**) GEM tissue concentrations. GEM concentrations in surrounding tissues following the implantation of the non-woven sheets in mice were measured at each time point. GEM concentrations released from the GEM-containing PLLA sheets at 1 h, 4 h, 1 d, 2 d, and 7 d were 89.6 ± 13.4, 21.5 ± 12.3, 1.1 ± 0.3, 1.3 ± 0.6, and 2.4 ± 0.8 μg/mL, respectively. In contrast, GEM concentrations released from the GEM-free PLLA sheets containing the GEM solution at 1 and 4 h were 132.4 ± 54.3 and 2.3 ± 0.8 μg/mL, respectively, but GEM was not detected after 1 d. (**b**) Diagram showing the transplantation process. Tumors were implanted intradermally, and non-woven sheets were implanted subcutaneously. GEM-free non-woven sheets were injected with GEM at the time of implantation. (**c**) Antitumor effects of the GEM-containing non-woven sheets. Mice were subcutaneously inoculated with Panc02 cells, and changes in tumor size were measured over time. Control group: Tumor size on days 0, 7, 14, 21, and 28 was 40.3 ± 12.1, 301.9 ± 78.2, 664.8 ± 157.4, 1801.2 ± 353.7, and 3118.3 ± 384.9 mm^3^, respectively (n = 6). Systemic administration group: Tumor size on days 0, 7, 14, 21, and 28 was 36.0 ± 2.3, 260.4 ± 16.7, 929.0 ± 121.2, 1835.3 ± 269.8, and 3588.9 ± 397.7 mm^3^, respectively (n = 4). Subcutaneous administration (GEM 1.0 mg) group: Tumor size on days 0, 7, 14, 21, and 28 was 38.0 ± 2.0, 283.5 ± 44.6, 744.4 ± 65.9, 2019.6 ± 257.3, and 3544.4 ± 556.9 mm^3^, respectively (n = 4). Non-woven PLLA/GEM (GEM 0.5 mg) sheet group: Tumor size on days 0, 7, 14, 21, and 28 was 34.9 ± 9.7, 151.8 ± 34.7, 326.5 ± 62.1, 923.0 ± 96.8, and 1384.5 ± 158.4 mm^3^, respectively (n = 4). Non-PLLA/GEM (GEM 1.0 mg) woven sheet group: Tumor size on days 0, 7, 14, 21, and 28 was 31.4 ± 7.6, 68.9 ± 24.0, 125.0 ± 45.5, 388.9 ± 98.6, and 702.2 ± 163.9 mm^3^, respectively (n = 7). Results are presented as the mean ± SEM. Significant differences were determined using Student’s *t*-test (* *p* < 0.05, ** *p* < 0.001, *** *p* < 0.005, n.s: Not significant).

GEM tissue concentrations in areas surrounding the non-woven sheet were measured in both groups, namely, that subcutaneously administered the GEM solution and that subcutaneously implanted GEM-containing non-woven sheets (Figure 4a,b).

After intradermally seeding tumor cells (Panc02) in mice, the preparations were administered and implanted to the mice, and tumor diameter was measured over a period of 28 d (Figure 4c). Each group consisted of four or more mice.

## 4. Discussion

We developed an electrospun biodegradable non-woven sheet for the controlled release of GEM. GEM is a standard anticancer drug used for pancreatic cancer treatment. It is a water-soluble antimetabolite; thus, it is not suitable for local administration in the peritoneal cavity [17]. PLLA was selected as the backing material polymer because of its biodegradability and biocompatibility [16,18]. In this study, we designed biocompatible GEM-containing nanofibers (GEM sheets) using electrospinning and evaluated their antitumor efficacy in murine pancreatic cancer models.

The scanning electron micrographs of non-woven fibers soaked in PBS showed that fiber diameter progressively decreased, but the shape of the fibers was preserved. Electrospinning of emulsions composed of a PLLA solution and GEM in the presence of HFIP as a catalyst yielded non-woven sheets with a mean fiber diameter of approximately 1–2 µm. As the GEM concentration increased, the fiber diameter progressively decreased because the dissolution of the water-soluble compound led to an increase in the ion concentration, which in turn increased the electrical conductivity. This increase in electrical conductivity resulted in a smaller fiber diameter, which in turn demonstrated similar characteristics to those reported previously [19]. There was a difference in fiber denaturation between the in vitro and in vivo experiments. In vivo, fiber diameter increased more than it did in vitro; in vivo, fiber fragmentation occurred over time, as it was blistered and cut into chunks. In vivo, PLLA fibers are degraded by proteinase K-catalyzed enzymes [20,21]. This difference in denaturation has a significant impact on the sustained release of GEM from the fibers.

In vitro, the cumulative release of GEM over 30 and 60 d from PLLA non-woven sheets containing 10 wt% GEM was approximately 35% and 36%, respectively, whereas that observed in vivo was 61% and 68%, respectively.

This long-term sustained release may be attributed to the fact that PLLA is a crystalline polymer. GEM was homogeneously dispersed in the solution and crystallized during fiber formation by field spinning. Differential scanning calorimetric measurements showed a GEM crystallinity of 35% (data not shown). In this crystallized state, GEM is suggested to exist in the amorphous region of the fiber [22,23]. GEM was initially released from the amorphous region close to the surface of the fiber (the initial burst), and that which was located in the amorphous region surrounded by the crystallized region was not released from the fiber within 60 d. In contrast, in vivo, hydrolysis of the crystallized region by proteinase K-catalyzed enzymes is thought to cause fiber disintegration, resulting in the sustained release of more GEM from the amorphous region surrounded by the crystallized region [24].

PLLA non-woven sheets containing more than 0.1 wt% GEM elicited antitumor effects against Panc02 cells (murine pancreatic cancer cell line) under both direct contact and non-contact conditions. The in vitro cytotoxicity evaluation showed that non-woven sheets containing GEM inhibited cell growth. As the non-woven sheets were set up as a scaffold material for the cells, to exclude the possibility of cytotoxicity being affected by contact between the cells and the non-woven sheets or GEM present on the surface, a cytotoxicity test with a transwell chamber was performed. It was established that cytotoxicity also occurred due to GEM release from the non-woven sheets. In direct contact conditions, cytotoxicity was high owing to the effect of direct exposure to the initial burst and the contact effect of the sheets, whereas under non-contact conditions, the effect of the initial burst was absent, and GEM cytotoxicity at 0.01 wt% was lower than that observed under contact conditions because its effects were due to diffusion alone. As a result, although cytotoxic activity was observed in non-woven sheets containing 0.01 wt% GEM under contact conditions, no cytotoxic activity was observed in these sheets under non-contact conditions. These results suggest that in vivo, non-woven sheets with low GEM concentrations only exhibit antitumor efficacy at their location of application. In addition, although NIH-3T3 has been shown to be sensitive to GEM [25], the cytotoxic effects of the sheets against NIH-3T3 cells were low even under contact conditions, suggesting that these effects do not affect normal cells at the location of application. These data show that PLLA non-woven sheets containing GEM possess a controlled release effect.

Next, we measured GEM concentrations in the subcutaneous tissues and blood of normal mice in which two pieces of non-woven sheets containing 10 wt% GEM were implanted or 2.0 mg of GEM solution was administered. The peak concentration of GEM in the blood of mice in the “non-woven sheet” group was approximately 1/100 that in the blood of mice in the aqueous group, with approximately 1 μg/mL GEM detected in mouse blood at least 7 d following sheet implantation (Supplementary Data; two pieces of 10 wt% GEM non-woven sheets and 100 µL of 20 mg/mL GEM solution). 

Based on previous findings, we evaluated the effects of the GEM non-woven sheets on normal tissues. In the cytotoxicity assay, 13.5 mg of 0.01 wt% GEM was divided into 24 wells, with each well containing 1.35 μg of GEM. In the GEM release assay, approximately 30% of GEM was released within 24 h of sheet setting. Considering these data, in the cytotoxic assay, the quantity of GEM in each of the 24 wells was 1.35 μg × 30% = 0.405 μg/well. The volume of medium per well was 500 μL; therefore, GEM concentration per well was 0.81 μg/mL. There was a slight difference between the concentration of GEM in mouse blood 24 h after the implantation of the GEM-containing non-woven sheets and that calculated from 0.01 wt% GEM in the in vitro cytotoxic assay at 24 h. As in vivo and in vitro metabolisms are different, it is difficult to make a simple comparison between them, but we speculate that a GEM concentration of 1.3 μg/mL per well (when the release rate was calculated based on the in vitro concentration curve in Figure 2a) is not significantly different from the in vivo blood concentration. Therefore, the implantation of two pieces of 10 wt% GEM-containing sheets in vivo corresponded to a concentration of 0.01 wt% GEM in the cytotoxic assay. In the cytotoxic assay, 0.01 wt% GEM was found not to exhibit cytotoxic effects against NIH-3T3 cells and thus is considered to have negligible effects on normal tissues.

In the in vivo experiment, there was an initial burst in the tissues into which 10 mg of 10 wt% GEM (1 mg of GEM per head)-containing sheets was implanted 1–4 h following sheet implantation, followed by a sustained GEM concentration within the range of 1.2 to 2.4 μg/g from day 1 to at least 7 d after sheet implantation. The concentrations of GEM in the GEM-containing non-woven sheets were approximately the same as those in the blood of mice administered double the dose of the GEM-containing non-woven sheets. It was demonstrated that a constant GEM concentration was sustained in the tissues into which the GEM-containing non-woven sheets were implanted for a relatively long period of time. In addition, in vivo therapeutic experiments showed that local administration of the GEM solution (20 mg/mL × 50 μL) exerted no antitumor effects, whereas significant and consistent antitumor effects were observed for a relatively long period of time when the GEM-containing non-woven sheets were implanted close to the tumor site. When GEM is applied intraperitoneally for the treatment of post-surgical local pancreatic cancer recurrence, the released GEM is immediately metabolized owing to its water-soluble nature; however, GEM contained in the non-woven sheets is expected to remain localized and be gradually released over time. As GEM is a time- and concentration-dependent anticancer drug, prolonged contact with tumor cells is essential for its antitumor effects, and our non-woven formulation is expected to be an effective tool in that respect [7,8,9]. Thus, as it is possible to maintain local GEM concentrations without increasing its blood concentrations, the clinical use of this drug may follow its implantation in the resection site following pancreatic cancer surgery. However, the limited opportunity for use, arising from being restricted to a local site, may be a disadvantage, just as GIGLIADEL is currently used in clinical practice for the treatment of malignant gliomas [26,27]. Therefore, we would like to consider a form that can be used for occasions other than during surgery, such as ordinary anticancer drugs in the future.

## 5. Conclusions

We successfully prepared GEM-containing PLLA non-woven sheets and demonstrated that they exhibit antitumor effects against mouse pancreatic cancer cells in vitro and in vivo. In addition, we measured the tissue concentrations of GEM released from the GEM-containing non-woven sheets and demonstrated that these sheets could contribute to the inhibition of tumor cell growth. As our results were obtained in murine subcutaneous tumor models, the evaluation of PLLA non-woven sheet safety when attached to the remnant of the pancreatic stump and need to control drug release in the transplantation environment are issues that will be addressed in the future for the possible application of this formulation in clinical practice.

## Data Availability

The data presented in this study are available on reasonable request from the corresponding author.

## Supplementary Materials

The following are available online at https://www.mdpi.com/article/10.3390/polym14010168/s1, Figure S1: two pieces of 10 wt% GEM non-woven sheets and 100 µL of 20 mg/mL GEM solution.
