# Supplementary figures and images for: Non-Woven Sheet Containing Gemcitabine: Controlled Release Complex for Pancreatic Cancer Treatment
Source: Polymers (Basel). 2022 Jan 1;14(1):168. doi: 10.3390/polym14010168 (PMC8747259; doi:10.3390/polym14010168)

Figure S1

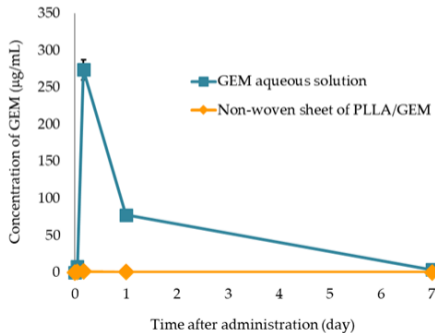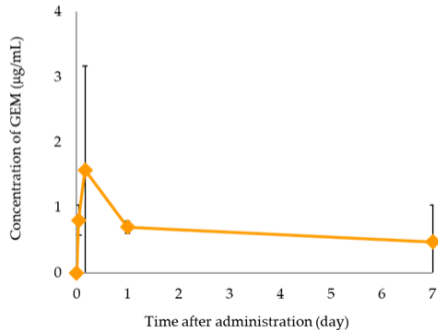

Supplement: Supplementary file 1 [file polymers-14-00168-s001.zip › polymers-1521027-SI.pdf]
